# Supplementary material for: COVID-19 Vaccine for Children: Determinants and Beliefs Contributing to Vaccination Decision of Parents in Germany 2021/2022
Source: Vaccines (Basel). 2023 Dec 23;12(1):20. doi: 10.3390/vaccines12010020 (PMC10820980; doi:10.3390/vaccines12010020)
Supplement: Supplementary file 1 [file vaccines-12-00020-s001.zip › vaccines-2741885-supplementary.pdf]

Table S1
Allocation of Participants by German Federal States

**Federal State in Germany**	***n***	**%**
Baden-Württemberg	325	13.5
Bayern	347	14.5
Berlin	101	4.2
Brandenburg	37	1.5
Bremen	11	0.5
Hamburg	71	3.0
Hessen	159	6.6
Mecklenburg-Vorpommern	16	0.7
Niedersachsen	262	10.9
Nordrhein-Westfalen	715	29.8
Rheinland-Pfalz	106	4.4
Saarland	22	0.9
Sachsen	65	2.7
Sachsen-Anhalt	28	1.2
Schleswig-Holstein	113	4.7
Thüringen	23	1.0

Table S2
Parents' Beliefs Contributing to Children COVID-19 Vaccination Uptake or Hesitancy

Categories	Beliefs	*n*	%
**Parents who are willing to have their children vaccinated** [1] (*n* = 1069)	Protect child/children from infection in every-day life (daycare, school...)	915	87.1
	Fear of a severe course	634	60.3
	Fear of "Long COVID-19"	875	83.3
	Chronically ill/immunocompromised child/children	116	11.0
	Child/children want/desire vaccination	392	37.3
	Regain more freedoms	442	42.1
**Parents who already approved the vaccination of their children prior to the official recommendation** (*n* = 863)	Achieve sufficient vaccination protection at an early stage.	770	89.3
	Reduce infections in daycare/school and avoid quarantine	765	88.7
	Regain more freedom	269	31.2
	Fear of a severe course	661	76.7
	Chronically ill/immunocompromised child/children	152	17.6
**Parents who refused to vaccinate their children** (*n* = 465)	I think that my child/children is/are too young	186	40.0
	I think that the vaccine is not well-studied	356	76.6
	I am not aware of the long-term effects	381	81.9
	I think my child/children would pass an infection without severe progression	357	76.8
	My child/children has/have an allergy to vaccine components, thrombosis-with-thrombocytopenia syndrome, or capillary leak syndrome	13	2.8
	Corona pandemic is an adult pandemic, which affects children less	223	48.0
	COVID-19 infection is less severe in children.	383	82.4
	My child/children do not belong to particularly vulnerable or pre-diseased groups	348	74.8
	My child/children refuse/refuse vaccination	109	23.4

[1] Parents who are willing to have their children vaccinated with and without the Standing Committee on Vaccination and who are undecided. Their children are not (yet) vaccinated (*n* = 1069)

Table S3
Determinants Stratified by Parents' COVID-19 Vaccination Willingness

Determinants	Parents' willingness for COVID-19 vaccination of children aged 5 to 11 years							
	Total (N = 2401)		Yes (*n* = 1714)		Undecided (*n* = 222)		No (*n* = 465)	
	n	%	*n*	%	*n*	%	*n*	%
**Sociodemographic**								
**Age** (N = 2390)								
< 39	1124	47.0	717	42.0	127	57.2	280	60.6
≥ 39	1266	52.9	989	58.0	95	42.8	182	39.4
**Gender**								
Female	2252	93.8	1614	94.2	212	95.5	426	91.6
Male	149	6.2	100	5.8	10	4.5	39	8.4
**Marital Status**								
Single	73	3.0	46	2.7	8	3.6	19	4.1
Married	2007	83.6	1461	85.2	192	86.5	354	76.1
In a relationship	223	9.3	135	7.9	17	7.7	71	15.3
Separated/divorced	93	3.9	69	4.0	5	2.3	19	4.1
Widowed	3	0.1	2	0.1	0	0.0	1	0.2
Other	2	0.1	1	0.1	0	0.0	1	0.2
**Level of education** (N = 2388)								
University degree	1367	57.2	1056	61.8	108	49.3	203	44.2
High school degree	315	13.2	211	12.3	26	11.9	78	17.0
Higher middle school degree	105	4.4	57	3.3	14	6.4	34	7.4
Lower middle school degree	597	25.0	384	22.5	71	32.4	142	30.9
Other forms of schooling	4	0.2	2	0.1	0	0.0	2	0.4
**Residence Area**								
100 000 residents	852	35.5	632	36.9	77	34.7	143	30.8
20 000 residents	629	26.2	423	24.7	64	28.8	142	30.5
5 000 residents	426	17.7	298	17.4	35	15.8	93	20.0
< 5 000 residents	494	20.6	361	21.1	46	20.7	87	18.7
**Mental health status**								
**DT**	5.70 [1]	2.654 [2]						
< 4	551	22.9	403	23.5	41	18.5	107	23.0
≥ 4	1850	77.1	1311	76.5	181	81.5	358	77.0
**GAD-7**	5.30 [1]	4.296 [2]						
< 5	1212	50.5	895	52.2	107	48.2	210	45.2
≥ 5	824	34.3	579	33.8	81	36.5	164	35.3
≥ 10	259	10.8	174	10.2	24	10.8	61	13.1
≥ 15	106	4.4	66	3.9	10	4.5	30	6.5
**PHQ-8**	5.60 [1]	4.338 [2]						
< 5	1132	47.1	821	47.9	93	41.9	218	46.9
≥ 5	864	36.0	627	36.6	80	36.0	157	33.8
≥ 10	290	12.1	189	11.0	37	16.7	64	13.8
≥ 15	88	3.7	59	3.4	10	4.5	19	4.1

≥ 20	27	1.1	18	1.1	2	0.9	7	1.5
**General vaccination attitudes**								
**Vaccination certificate (parents)**								
Complete	1891	78.8	1558	90.9	182	82.0	151	32.5
Partial complete	309	12.9	146	8.5	35	15.8	128	27.5
Incomplete	195	8.1	10	0.6	5	2.3	180	38.7
So far, the COVID-19 vaccination is the only vaccination I have ever received.	6	0.2	0	0.0	0	0.0	6	1.3
**Vaccination certificate (children)**								
Complete	2048	85.3	1619	94.5	195	87.8	234	50.3
I do not know	9	0.4	2	0.1	1	0.5	6	1.3
Incomplete	288	12.0	91	5.3	25	11.3	172	37.0
My child has not received any childhood vaccinations.	56	2.3	2	0.1	1	0.5	53	11.4
**Importance of immunizations as protection against other infections**	84.89 [1]	26.464 [2]						
< 50	224	9.33	7	0.4	6	2.7	211	45.4
≥ 50	2177	90.67	1707	99.6	216	97.3	254	54.6
**COVID-19 vaccination of parents**								
Yes	2084	86.8	1713	99.9	214	96.4	157	33.8
No	317	13.2	1	0.1	8	3.6	308	66.2
**SARS-CoV-2 politics perceptions**								
**COVID-19 related fear**	5.30 [1]	1.683 [2]						
< 5	505	21.0	109	6.4	54	24.3	342	73.5
≥ 5	1896	79.0	1605	93.6	168	75.7	123	26.5
**Subjective level of information**	6.00 [1]	1.082 [2]						
< 5	247	10.3	51	3.0	26	11.7	170	36.6
≥ 5	2154	89.7	1663	97.0	196	88.3	295	63.4
**Governmental trust**	3.90 [1]	1.199 [2]						
< 5	1805	75.2	1197	69.8	163	73.4	445	95.7
≥ 5	596	24.8	517	30.2	59	26.6	20	4.3
**Pressure perception by COVID-19 vaccination campaigns**	26.50 [1]	37.741 [2]						
< 50	1763	73.4	1590	92.8	116	52.3	57	12.3
≥ 50	638	26.6	124	7.2	106	47.7	408	87.7
**Experienced restrictions of the social environment**								
Very little	290	12.1	271	15.8	11	5.0	8	1.7
Little	881	36.7	744	43.4	94	42.3	43	9.2
Neither	382	15.9	326	19.0	31	14.0	25	5.4
Strongly	579	24.1	340	19.8	72	32.4	167	35.9
Very strong	269	11.2	33	1.9	14	6.3	222	47.7
**Engagement against protective measures**								
Never, and do not plan to	1905	79.3	1577	92.0	190	85.6	138	29.7

Never, but plan to do so	105	4.4	10	0.6	8	3.6	87	18.7
Once	57	2.4	19	1.1	6	2.7	32	6.9
Several times	228	9.5	66	3.9	16	7.2	146	31.4
Regularly	106	4.4	42	2.5	2	0.9	62	13.3
**Attitude towards mandatory measles vaccine**								
Fully adequate	1516	63.1	1366	79.7	100	45.0	50	10.8
Adequate	433	18.0	294	17.2	76	34.2	63	13.5
Neutral	158	6.6	37	2.2	34	15.3	87	18.7
Exaggerated	154	6.4	16	0.9	11	5.0	127	27.3
Completely overdone	140	5.8	1	0.1	1	0.5	138	29.7

	**Pressure perception by COVID-19 vaccination campaigns**			
**Attitude towards mandatory measles vaccine**	< 50 (n = 1763)		≥ 50 (n = 638)	
Fully adequate	1359	77.1	157	24.6
Adequate	314	17.8	119	18.7
Neutral	44	2.5	114	17.9
Exaggerated	26	1.5	128	20.1
Completely overdone	20	1.1	120	18.8

[1] Mean score

[2] Standard deviation
